# Improving the Theranostic Potential of Magnetic Nanoparticles by Coating with Natural Rubber Latex for Ultrasound, Photoacoustic Imaging, and Magnetic Hyperthermia: An In Vitro Study

**DOI:** 10.3390/pharmaceutics16111474

**Published:** 2024-11-19

**Authors:** Thiago T. Vicente, Saeideh Arsalani, Mateus S. Quiel, Guilherme S. P. Fernandes, Keteryne R. da Silva, Sandra Y. Fukada, Alexandre J. Gualdi, Éder J. Guidelli, Oswaldo Baffa, Antônio A. O. Carneiro, Ana Paula Ramos, Theo Z. Pavan

**Affiliations:** 1 Department of Physics, FFCLRP, University of São Paulo, Av. Bandeirantes 3900, Ribeirão Preto 14040-901, São Paulo, Brazil; thiagotiburcio37@usp.br (T.T.V.); saeideh.arsalani@utsouthwestern.edu (S.A.); setubalmateus@usp.br (M.S.Q.); guilherme.santos.fernandes@usp.br (G.S.P.F.); guidelli@usp.br (É.J.G.); baffa@usp.br (O.B.); adilton@usp.br (A.A.O.C.); 2 UT Southwestern Medical Center, Biomedical Engineering Department, Dallas, TA 75235-7323, USA; 3 Department of BioMolecular Sciences, FCFRP, University of São Paulo, Av. Professor Doutor Zeferino Vaz, sn, Ribeirão Preto 14040-901, São Paulo, Brazil; keteryne@alumni.usp.br (K.R.d.S.); sfukada@usp.br (S.Y.F.); 4 Department of Physics, Federal University of São Carlos, Rod. Washington Luiz, km 235, São Carlos 13565-905, São Paulo, Brazil; gualdi@df.ufscar.br; 5 Department of Chemistry, FFCLRP, University of São Paulo, Av. Bandeirantes 3900, Ribeirão Preto 14040-901, São Paulo, Brazil; anapr@ffclrp.usp.br

**Keywords:** magnetic nanoparticles, natural rubber latex, theranostics, ultrasound, magnetomotive ultrasound, photoacoustic imaging, magnetic hyperthermia

## Abstract

Background/Objectives: Magnetic nanoparticles (MNPs) have gained attention in theranostics for their ability to combine diagnostic imaging and therapeutic capabilities in a single platform, enhancing targeted treatment and monitoring. Surface coatings are essential for stabilizing MNPs, improving biocompatibility, and preventing oxidation that could compromise their functionality. Natural rubber latex (NRL) offers a promising coating alternative due to its biocompatibility and stability-enhancing properties. While NRL-coated MNPs have shown potential in applications such as magnetic resonance imaging, their effectiveness in theranostics, particularly magnetic hyperthermia (MH) and photoacoustic imaging (PAI), remains underexplored. Methods: In this study, iron oxide nanoparticles were synthesized via coprecipitation, using NRL as the coating agent. The samples were labeled by NRL amount used during synthesis: NRL-100 for 100 μL and NRL-400 for 400 μL. Results: Characterization results showed that NRL-100 and NRL-400 samples exhibited improved stability with zeta potentials of −27 mV and −30 mV, respectively and higher saturation magnetization values of 79 emu/g and 88 emu/g of Fe_3_O_4_. Building on these findings, we evaluated the performance of these nanoparticles in biomedical applications, including magnetomotive ultrasound (MMUS), PAI, and MH. NRL-100 and NRL-400 samples showed greater displacements and higher contrast in MMUS than uncoated samples (5, 8, and 9 µm) at 0.5 wt%. In addition, NRL-coated samples demonstrated an improved signal-to-noise ratio (SNR) in PAI. SNR values were 24.72 (0.51), 31.44 (0.44), and 33.81 (0.46) dB for the phantoms containing uncoated MNPs, NRL-100, and NRL-400, respectively. Calorimetric measurements for MH confirmed the potential of NRL-coated MNPs as efficient heat-generating agents, showing values of 43 and 40 W/g for NRL-100 and NRL-400, respectively. Conclusions: Overall, NRL-coated MNPs showed great promise as contrast agents in MMUS and PAI imaging, as well as in MH applications.

## 1. Introduction

Magnetic nanoparticles (MNPs) hold great promise in diverse biomedical applications, including targeted drug delivery, contrast agents for imaging techniques [1,2,3,4], and magnetic hyperthermia (MH) for tumor treatment [4,5,6]. For example, iron oxide nanoparticles (IONPs), such as maghemite (γ-Fe_2_O_3_) and magnetite (Fe_3_O_4_), are commonly used due to their desirable magnetic properties, such as superparamagnetic behavior, small sizes, and low toxicity to the human body [7]. However, MNPs can exhibit colloidal instability, necessitating their functionalization using surface binders such as polyethylene glycol (PEG) [8,9], chitosan [10], gelatin [11], bovine or human serum albumin (BSA and HSA) [12,13], noble metals [14], and natural rubber latex (NRL) [15]. Among these, NRL, extracted from *Hevea brasiliensis* (rubber tree) native to the Amazon River basin [16], exists as a colloidal suspension. This suspension is obtained through manual extraction and primarily consists of lipid and protein-based particles encapsulating a hydrophilic core composed of long poly(cis-1,4-isoprene) chains. NRL offers multiple advantages for biomedical applications, enabling nanoparticle functionalization, size control, and enhanced colloidal stability, with surface modifications for binding various molecules. This versatility facilitates applications such as controlled drug release in bone regeneration, tissue repair, vascular prosthetics, and as a biocompatible coating for materials such as silver, gold, and IONPs [15,16,17,18,19,20].

These coating agents play an important role in stabilizing MNPs and functionalizing their surfaces for specific applications [21,22,23], providing essential benefits in biomedical uses due to their biocompatibility. These coatings help prevent large particle aggregates, reduce precipitation in aqueous environments, and lower the risk of vascular blockage. They also preserve the structural integrity of nanoparticles, increase their resistance to biodegradation in biological systems, and enhance their magnetic properties and biocompatibility. Together, these improvements make MNPs highly suitable for biomedical targeting and theranostic applications, particularly in tumor treatment [24,25,26,27,28,29].

The concept of theranostics represents a significant advance in medicine, as it involves the integration of diagnostic and therapeutic functionalities in a single platform, which can be applied simultaneously or sequentially. This approach enables personalized treatment, adjusting therapeutic interventions based on real-time diagnostic responses, leading to better patient results and more efficient use of healthcare resources [30,31]. Nanoparticle-based thermal therapy exemplifies this approach by combining imaging capabilities with heat generation to target and treat diseased tissue. These nanoparticles can be engineered to accumulate selectively in tumor sites, where they can be visualized using imaging modalities such as magnetic resonance imaging (MRI) or ultrasound [32]. Upon activation by an external energy source, such as a magnetic field or light, the nanoparticles produce localized hyperthermia for cancer treatment [33]. This dual functionality enhances the precision of treatments and allows for continuous monitoring and adjustment, improving patient outcomes and reducing side effects.

Ultrasound is a widely used diagnostic imaging technique in medical practice, but it has limitations, such as operator dependency and lower image contrast. For example, the contrast and resolution of ultrasound images are not sufficient for efficiently detecting nanoparticles within tissue. To address this challenge, various techniques have been proposed to monitor specific physical effects resulting from the interaction of nanoparticles with an external stimulus applied to the target area [34]. One example is the magnetomotive ultrasound (MMUS) technique, developed to localize MNPs using ultrasound. In this method, external low-frequency (<100 Hz) magnetic fields induce tissue motion in regions labeled with magnetic materials, and ultrasound images are used to track this motion with the ultimate goal of localizing MNPs [35,36]. The technique has been explored for various applications, including detecting MNPs in mice tumors and rat lymph nodes [37,38], observation of MNP endocytosis in living cells [39], and the imaging of blood clots [40]. Recently, our group proposed a theranostic platform that combines MMUS with MH to enable real-time detection of MNPs and monitor therapeutic outcomes using ultrasound [41].

Photoacoustic imaging (PAI) is an imaging modality that combines the high contrast of optical imaging with the detailed resolution of ultrasound imaging. This technique generates real-time functional images without relying on ionizing radiation, ensuring good mobility and cost-effectiveness [42,43]. PAI has a wide range of biomedical applications, for example, tomographic visualization of skin and superficial organs using laser-induced photoacoustic microscopy, breast and skin imaging for cancer detection, and gastrointestinal imaging [43]. MMUS has been combined with PAI to enhance sensitivity and create magnetomotive photoacoustic (MMPA) imaging. In MMPA, ultrasound and photoacoustic images are captured while applying an external magnetic field. In this context, modified MNPs with increased optical absorption have been explored to improve the efficacy of the technique [44].

On the other hand, applying a higher-frequency (100 kHz–1 MHz) alternating (AC) magnetic field to tissue loaded with MNPs leads to heat generation. In the field of biomedicine, this approach has predominantly been employed as a method for cancer treatment [45,46]. In the context of hyperthermia in cancer treatment, IONPs are used as intra-tumor agents under an AC magnetic field applicator, reaching a temperature range between 40 and 43 °C, which is safe for the patient [45]. In this range, the selective effect of heat promotes the damage or death of cancer cells due to the influence of high temperature [45,46]. The heating efficiency, often referred to as specific loss power (SLP), is closely related to the thermal losses of the MNPs when subjected to an AC magnetic field and is determined by the area of the MNPs hysteresis loop [47]. In addition to the amplitude and frequency of the applied AC magnetic field, SLP also depends on the saturation magnetization, size, concentration, and effective anisotropy of the MNPs [48].

NRL-coated MNPs have been investigated for applications such as contrast agents in MRI [49] and for enhancing shear wave dispersion in MMUS [50]. These studies have shown promising results, suggesting that NRL-coated MNPs could be a valuable tool for nanotheranostics. However, their performance in MH and PAI has not been thoroughly explored, and the mechanisms behind their enhanced performance in MMUS are not yet fully understood. Therefore, the present paper aims to evaluate NRL-coated MNPs as contrast agents for PAI and as heating mediators for MH. In addition, to gain a comprehensive understanding of these MNPs in MMUS, we conducted extensive experiments using tissue-mimicking phantoms with varying concentrations and characterized their magnetophoretic behavior. The MNPs were synthesized using a coprecipitation method with varying amounts of NRL, and their physical properties were characterized using various techniques. Finally, biological studies were performed to assess cytotoxicity using the mouse melanoma cell line (B16-F10).

## 2. Materials and Methods

### 2.1. Chemical Reagents

The chemical reagents iron chloride hexahydrate (FeCl_3_·6H_2_O) and iron chloride tetrahydrate (FeCl_2_·4H_2_O) were acquired from Sigma-Aldrich (St. Louis, MO, USA). Ammonium hydroxide (NH_4_OH; 30%) and hydrochloric acid (HCl; 38%) were purchased from Ecibra (São Paulo, SP, Brazil) and Synth (São Paulo, SP, Brazil), respectively. NRL used in this research comprises an extract from multiple clones of *Heveas brasiliensis* and was supplied by BDF Comercio de Produtos Agricolas Ltd. (Guarantã, SP, Brazil).

#### 2.1.1. Preparation of Fe_3_O_4_ NPs

The coprecipitation method was employed to synthesize MNPs. For this, a 1 mol/L FeCl_3_ solution was prepared by dissolving 6.75 g of FeCl_3_·6H_2_O in 25 mL of deionized water. Subsequently, a 2 mol/L FeCl_2_ solution was prepared by dissolving 3.97 g of FeCl_2_·4H_2_O in 10 mL of an aqueous HCl solution (5.45 M). In a volumetric flask, 4 mL of the FeCl_3_·6H_2_O solution and 1 mL of the FeCl_2_·4H_2_O solution were mixed (molar ratio of 2:1, the total iron concentration was 1.284 M), keeping the solution under mechanical stirring at 90 °C. Then, a 1.28 M NH_4_OH (2.5 mL of NH_4_OH in 47.5 mL of deionized water) solution was added to the flask containing the mixture of ferric salts at 90 °C, using an infusion pump with a flow rate of 6 mL/min. After adding the NH_4_OH solution, the color of the mixture changed to black, indicating the formation of MNPs. The resulting suspension was subjected to a one-hour treatment in an ultrasonic bath to prevent agglomeration. MNPs were precipitated with a magnet and washed with pure water several times until a neutral pH was reached. Finally, the solid was dried in a vacuum oven at 32 °C for 24 h, obtaining black powder.

#### 2.1.2. Preparation of Nanoparticles Coated with Latex

The method used to prepare the NRL-coated MNPs is similar to that used to prepare the uncoated NPs. For this, 100 or 400 µL of NRL were added to the 1.28 M NH_4_OH solution (it was observed that the pH of the ammonium hydroxide solution remained unchanged when we added 100 and 400 µL of NRL) before addition to the ferric mixture [15]. Following the same procedure described for the preparation of Fe_3_O_4_ NPs, samples were then categorized according to the amount of NRL added during synthesis. The sample prepared with 100 μL of NRL was labeled NRL-100, while the sample with 400 μL of NRL was labeled NRL-400. The amount of NRL used in the synthesis was based on previous [15,50].

### 2.2. Characterization of MNPs

Different techniques characterized the NPs. The hydrodynamic diameter and zeta potential were determined via dynamic light scattering (DLS) using the Zeta-Sizer Nano from Malvern Instruments (Malvern, UK). DLS measurements (Z-average and zeta potential) were carried out in deionized water with a pH of approximately 6.4. The crystalline properties and phase identification of these materials were obtained using the X-ray diffraction (XRD) technique. For this, a Bruker Siemens AXS D5005 X-ray diffractometer with Cu Kα radiation (λ = 1.5406 Å) was employed in the range of 10° < 2θ < 70°. The MAUD software (https://luttero.github.io/maud/) was used to determine the peak positions, perform adjustments, and carry out refinements.

The morphology of NPs was investigated by transmission electron microscopy (TEM). TEM images were obtained with a JEOL-JEM-100 CXII instrument (Peabody, MA, USA). To this end, a drop of colloidal dispersion was dried on a copper grid covered with a conductive polymer. The ImageJ software (https://imagej.net/ij/) was used to determine the size distribution of NPs using the TEM images. The average diameter of the NPs was determined by counting approximately 150 particles for each sample. The Origin Pro 8.5 software was used to create histograms for statistical analysis.

The chemical groups present in the NPs were investigated using Fourier transform infrared (FTIR) spectroscopy coupled to an attenuated total reflectance (ATR) accessory based on ZnSe (Shimadzu IR-Prestige, Kyoto, Japan). Spectra were obtained from 4000 and 500 cm^−1^. UV-visible absorption spectra were obtained in the 200 to 800 nm range using the Amersham Ultrospec 2100 Pro spectrophotometer (Amersham, UK).

Thermogravimetric analysis (TGA) of the powdered samples was conducted from 25 to 900 °C under an oxidizing atmosphere with a heating rate of 10 °C/min using TGA/DTA/DSC equipment (Model instruments SDTQ 600-TA, New Castle, DE, USA).

The magnetic properties of the samples were studied using a Lake Shore vibrating-sample magnetometry (VSM) model 7400 (Westerville, OH, USA) with a sensitivity of 10^−7^ emu and a maximum field amplitude of 2 T. The dried samples had a total weight of around 50 mg, but the magnetization curves were normalized by the mass of iron oxide, i.e., the mass of the latex was subtracted. Magnetization measurements were performed as a function of the M(H) field at a temperature of approximately 300 K. A magnetic separation system (SEPMAG, Barcelona, Spain) equipped with a magnetic field gradient of 15 T m^−1^ was employed to investigate magnetophoretic behavior, specifically the movement of MNPs in an inhomogeneous magnetic field, within the analyzed samples.

### 2.3. Cell Culture

To evaluate the toxicity of the NPs to cancer cells, an in vitro cell viability study was performed using a mouse melanoma model cell line (B16-F10). The cells were cultured in Dulbecco’s Modified Eagle’s Medium (DMEM) high-glucose medium (Sigma, Aldrich, Inc., St. Louis, MO, USA), supplemented with 10% fetal bovine serum (GIBCO, Grand Island, NY, USA) and 1% penicillin/streptomycin (GIBCO, Grand Island, NY, USA) in culture conditions at 37 °C and 5% CO_2_.

For this study, B16-F10 cells were seeded in 96-well plates at a density of 1 × 10^4^ cells per well. After allowing attachment for 24 h, the medium was replaced with different concentrations of NPs (1, 5, 10, 25, 50, and 100 µg/mL) and the cell viability was assessed. According to the manufacturer’s instructions, after 24 and 48 h of treatment using the CellTiter-Glo reagent. Control cells received an equivalent amount of diluent (PBS). Each test was conducted using biological and experimental triplicate. The luminescence was measured using the SpectraMax L microplate reader Software (https://www.moleculardevices.com/products/microplate-readers/luminescence-readers/spectramax-l-luminescence-reader, https://support.moleculardevices.com/s/article/SpectraMax-Microplate-Reader-User-Manual-Download-Page). The viability values of the treated cells were expressed as a percentage of those of the corresponding control cells.

### 2.4. Tissue-Mimicking Phantoms

Cylindrical tissue-mimicking phantoms containing hemispherical inclusions labeled with MNPs were produced to evaluate their performance as contrast agents for MMUS and PAI. The phantoms were prepared by mixing gelatin (Bloom 250, Gelita, São Paulo, Brazil) and agar (bacteriological CAT RM026 provided by Himedia, Thane, India) in water at concentrations of 5% and 2%, respectively [50]. The resulting mixture was heated to 90 °C to achieve a homogeneous and clear solution without visible trapped air bubbles [51,52].

To manufacture the inclusion, the NPs were subsequently added to the gelatin/agar solution at 50 °C, and formaldehyde was introduced at 5% of the gelatin mass to increase both the melting point and the elastic modulus. The molten mixture was poured into a hemispherical inclusion mold (20 mm in diameter and height) and refrigerated for 24 h to ensure consistent NPs distribution. Twelve inclusions were fabricated, each with a distinct concentration of MNPs (1, 0.75, 0.50, and 0.25 wt.%). Additionally, two extra inclusions were made for each type of NP and concentration, bringing the total number of phantoms to thirty-six. The subsequent step involved the fabrication of the gelatin/agar phantom background. The process was similar for preparing the inclusions, but this time, the gelatin was mixed with 2 wt.% agar to increase stiffness. Once it reached an ambient temperature of 35 °C, the solution was poured into a cylindrical mold with a diameter of 5 cm and a height of 2.5 cm. The phantom was then refrigerated for 24 h.

### 2.5. MMUS Experimental Setup

**Figure 1:**
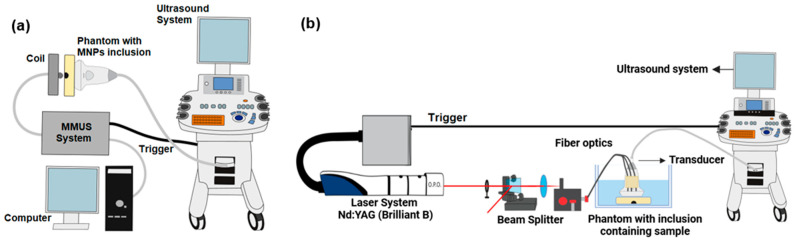
(**a**) Schematic representation of the experimental setup for MMUS imaging experiments. (**b**) Schematic representation of the PAI system using the Nd:YAG laser as the excitation source and the ultrasound imaging system to acquire the data.

The MMUS experimental setup included a coil with 130 turns, an inner diameter of 22 mm, an inductance of 114 µH, and a DC resistance of 218 mΩ. A 20 mm diameter steel core with a coercivity of 20 A/m was inserted in the center of the coil to enhance and focus the magnetic field. The interaction between the magnetic field and MNPs induced a magnetic force, resulting in vibration within the phantom [44]. A pulsed magnetic field was employed using a homemade system described [53,54] with a 6 ms duration. The amplitude of the magnetic field in the region containing the NPs, 2 mm from the tip of an iron core of the coil, was 0.1 T (measured by a GlobalMag, TMAG-1T, gauss meter). For ultrasound imaging, a Sonix RP ultrasound scanner (Ultrasonix, Richmond, BC, Canada) equipped with a linear ultrasound transducer (L14-5/38) operating at 9.5 MHz was used. The transducer was positioned opposite to the coil during data acquisition. Ultrasound data were acquired using a SonixDAQ (Ultrasonix, Richmond, BC, Canada) to achieve a high frame rate of 4 kHz, ensuring accurate sampling of the induced displacement. The synchronization between ultrasound acquisition and magnetic excitation was achieved using LabVIEW. The MMUS images were generated by detecting the displacements induced in MNP-labeled regions of the phantom. A specialized cross-correlation algorithm was applied to successive ultrasound echo signals acquired during magnetic field application [55]. The schematic representation of the MMUS experimental setup is shown in Figure 1a.

### 2.6. PAI Setup

The PAI system comprised an Nd:YAG laser (Brilliant B, Quantel Laser, Les Ulis, France) connected to a second harmonic generator module, which pumped an optical parametric oscillator (OPO) (MagicPRISM OPO, Opotek, Carlsbad, CA, USA). OPO is synchronizable in the 680–950 nm range for the signal function and 1200–2000 nm for the idler function. A 600 nm high-pass optical filter (FELH0600, Thorlabs Inc., Newton, NJ, USA) was used to eliminate contamination of a 532 nm component [56]. The resulting output beam was guided through a trifurcated optical fiber bundle (77,536, Newport) to illuminate the sample. A beam splitter diverted a fraction of the energy beam to record the energy level of each pulse. Each image was then normalized to account for pulse-to-pulse energy variation. A custom 3D-printed holder (ZMorph 2.0 SX, ZMorph, Wroclaw, Poland) was designed to connect the optical fiber bundle to the ultrasound transducer securely. The laser operated at a pulse repetition rate of 10 Hz and 750 nm optical wavelength. For image acquisition, a linear array transducer (L14-5/38, Ultrasonix) connected to an ultrasound machine (SonixOP, Ultrasonix) with a parallel acquisition module (SonixDAQ, Ultrasonix) operating with a sampling frequency of 40 MHz was used. The synchronization was achieved using the output signal of the laser (Q-Switch), which triggered the ultrasound device to initiate the acquisition of the photoacoustic signals [57]. The phantoms and transducer were placed in a water tank to acquire the photoacoustic images, as depicted in the schematic representation of the PAI setup in Figure 1b. For a more detailed description of the PAI system used in this study, the reader is referred to [57].

To quantitatively assess image quality, the signal-to-noise ratio (SNR) and contrast metrics were calculated using Equations (1) and (2):

(1) $$SNR=20\cdot \;{log}_{10}\left(\frac{\mu_{i}}{\sigma_{0}}\right)\;[dB],$$

(2) $$contrast=20\;\cdot {log}_{10}\left(\frac{\mu_{i}}{\mu_{0}}\right)\;[dB],$$

where $\mu_{i}$ and $\mu_{0}$ are the signal amplitudes averaged over a region of interest (ROI) defined around the inclusion and the background, respectively and $\sigma_{0}$ is the background ROI standard deviation [58]. The ROIs were defined consistently at the same depth and with uniform size.

### 2.7. MH Experiments

The ability of the MNPs to dissipate heat was evaluated through calorimetric measurements. MNPs were dispersed in water and exposed to an external alternating magnetic field using a homemade MH system [59]. The applied magnetic field had an amplitude of 100 Oe with a frequency of 137 kHz. A solenoid coil (14 mm in diameter and 87 mm in height) capable of generating a homogeneous field in a volume of 6.8 cm^3^ was used, ensuring that the entire sample was immersed in a region of uniform field [59]. Eppendorf tubes (eliminating any significant thermal influence during measurements), insulated with polystyrene foam, were positioned inside the solenoid coils. Real-time temperature measurements were taken using a fiber optic thermometer (Qualitrol NOMAD-Touch Portable Fiber Optic Monitor, Québec, QC, Canada) inserted directly into the nanoparticle solution, making it possible to obtain the measurement exclusively from the sample, with an accuracy of ±0.1 °C. The specific loss power (SLP) was estimated using the following equation.

(3) $$SLP=\frac{1}{m_{np}}C_{susp}\frac{∆T}{∆t},$$

where *m_np_* is the mass of MNPs, *C_susp_* is the thermal capacity of the suspension, Δ*T* the temperature variation, and *t* is the time.

## 3. Results and Discussion

### 3.1. Characterization of MNPs

The hydrodynamic diameter of MNPs was determined using DLS for pure NRL, NRL-coated, and uncoated Fe_3_O_4_ NPs. The Z-average values obtained for the colloidal suspension of NRL was 111 ± 33 nm, similar to that reported by other researchers [60,61]. For the MNPs, the values obtained were 53 ± 1, 53 ± 1, and 56 ± 2 nm for the uncoated MNPs, NRL-100 and NRL-400, respectively. Notably, the Z-average values of latex-coated MNPs were larger than those of the uncoated ones. In addition, the size of the NPs increased with the amount of NRL added, and there may be a correlation with the larger Z-average of the rubber molecules [15]. Refer to Figure S1 in the Supplementary Material for detailed hydrodynamic diameter results from DLS.

The surface charge of the samples was evaluated by measuring the zeta potential for the pure NRL, NRL-coated, and uncoated Fe_3_O_4_ NPs. A negative zeta potential value was obtained for NRL (−35 mV). According to Sansatsadeekul et al. [62], NRL zeta potential (obtained from Hevea brasiliensis) exhibits a negative value in the pH range from 4 to 12. The negative charges are attributed to the ionization of carboxylic acid groups at the surface of the rubber particles. These particles, composed of phospholipids, carbohydrates, proteins, and metal ions, collectively contribute to the overall charge, resulting in the highest zeta potential value [62]. Researchers in [62] obtained a zeta potential of −35 mV for NRL at pH 7 in accordance with the result obtained in this study. The zeta potential obtained for the uncoated MNPs, NRL-100 and NRL-400, were −15, −28, and −30 mV, respectively. Variation in zeta potential is a result of surface modification and is indicative of NPs stability. Electrostatically stabilized NPs typically exhibit values higher than ±30 mV [63,64]. Furthermore, the stability of these NPs is crucial information for biomedical applications, as it directly impacts their behavior—how they interact, disperse, and maintain their properties within biological systems. Based on this information, it can be inferred that NRL-coated NPs offer enhanced stability compared to uncoated ones, indicating their potential suitability for robust biomedical applications.

**Figure 2:**
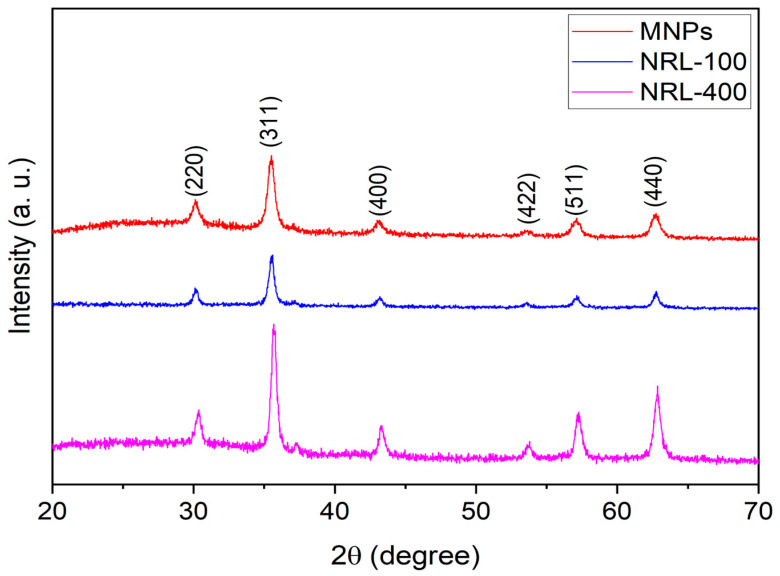
Diffraction patterns of uncoated and coated MNPs with different amounts of NRL. The crystallite size (d) was determined using the Rietveld method.

The structural properties and the average size of the crystallites were determined using X-ray diffraction. Figure 2 shows the diffraction of the samples. Peaks corresponding to those reported in the crystallographic chart CIF-10110325, specifically (220), (311), (400), (422), (511), and (440), indicative of the magnetite and maghemite structure. The experimental diffraction results were refined using the Rietveld method, which confirmed cubic symmetry and Fd-3m space group for all samples. The samples exhibited a lattice parameter close to 8.4 Å and different crystallite sizes 9, 16, and 20 nm for the uncoated MNPs, NRL-100 and NRL-400, respectively.

**Figure 3:**
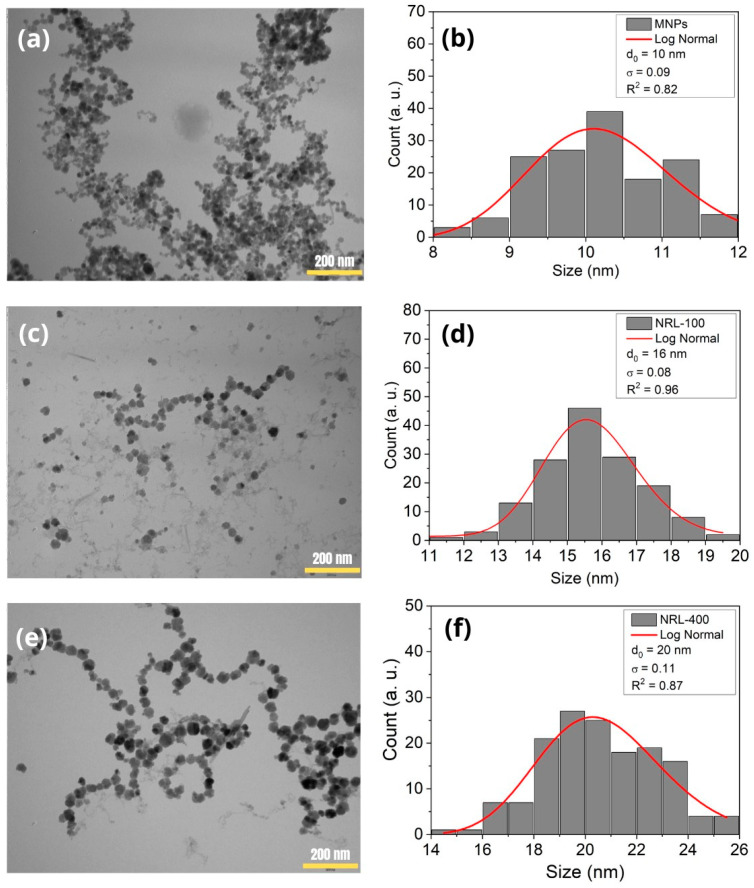
TEM images and histograms of the particle size distribution of uncoated MNPs (**a**,**b**), NRL-100 (**c**,**d**), and NRL-400 (**e**,**f**). Scale bar corresponds to 200 nm.

The TEM images shown in Figure 3a,c,e reveals the spherical shapes of the MNPs. Figure 3b,d,f shows histograms of the particle size distribution. The average diameters obtained for the uncoated MNPs, NRL-100, and NRL-400 were 10, 16, and 20 nm, respectively.

NRL-100 and NRL-400 NPs exhibited an increase in their average diameter compared to uncoated MNPs. This effect can be attributed to the polymer molecules, which act as a bridge between the particles, promoting their aggregation and consequently increasing the average diameter [65]. A comparison can be established between the results obtained by DLS and TEM since both techniques revealed a similar trend in size variation between the MNPs, NRL-100, and NRL-400. The difference between the techniques lies in the fact that DLS measures the hydrodynamic diameter, which includes not only the core of the nanoparticles but also any solvent layer or molecules adsorbed on the surface [66,67,68]. The NRL coating cannot be observed in TEM images due to the low electron density of the organic molecules composing the material. The polymer becomes transparent, while the magnetite particles absorb the electron beam, appearing as dark spots in the images. The average diameter determined by TEM was similar to the crystallite size determined by the Rietveld method in the XRD analysis, indicating that the particles are composed of single crystals. For more information on elemental analysis obtained through Energy-dispersive X-ray spectroscopy (EDS) and Scanning Electron Microscopy (SEM) images, see Figure S2 in the Supplementary Material.

**Figure 4:**
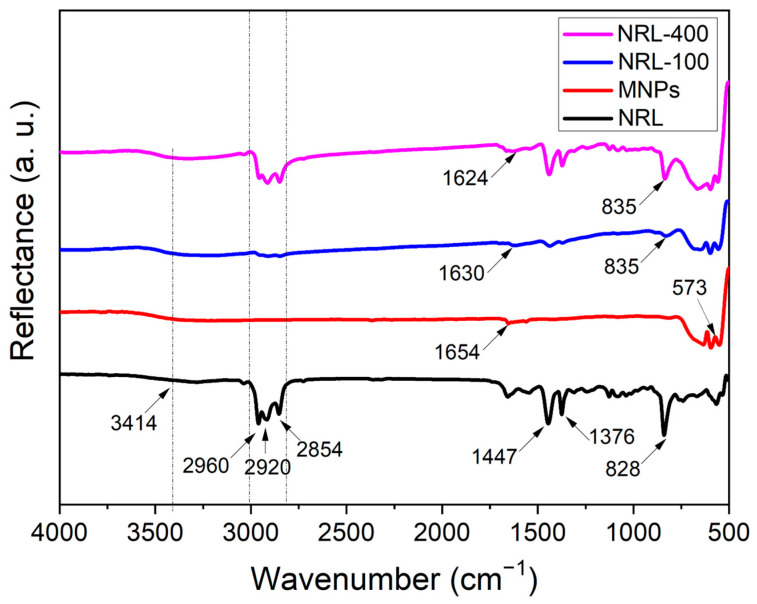
ATR spectra of NRL, uncoated MNPs, and MNPs coated with different amounts of NRL.

The FTIR-ATR technique was employed to assess the chemical groups present in the samples. Spectra were acquired from 4000 to 500 cm^−1^, confirming the adsorption of NRL on the surface of Fe_3_O_4_ NPs [15]. Figure 4 shows the spectra obtained for the NRL colloidal suspension and uncoated MNPs, NRL-100, and NRL-400. Peaks corresponding to chemical groups present in the NRL can be observed in the FTIR spectra of the NPs, as indicated by arrows in Figure 4.

Two absorption peaks at approximately 3414 cm^−1^ and 1624 cm^−1^ indicate the presence of O-H stretching vibrations and H-O-H scissoring, respectively. These hydroxyl groups could be attributed to the hydroxyl groups in the molecules that compose NRL or the presence of residual water on the surface of the NPs. According to Arsalani [15], the bands at 2960 and 834 cm^−1^ are associated with the C-H elongation. The peaks at 2920 and 2860 cm^−1^ are related to the symmetric and asymmetric elongation of the CH_3_ group, and the peaks at 1447 and 1376 cm^−1^ are related to the symmetric and asymmetric bending of the same group. The NRL spectrum exhibited a peak at approximately 827 cm^−1^ associated with C-H groups. This peak is shifted to 834 and 835 cm^−1^ in the spectra of samples NRL-100 and NRL-400, respectively. In addition, as the amount of NRL increased, there was a notable enhancement in the intensity of the peaks in the regions of 2960, 2918, and 2854 cm^−1^. According to Arsalani [15], the existence of C-H groups and the variations in intensity and position of these bands across all spectra of NPs with latex suggest the functionalization of MNPs (Fe_3_O_4_) with cis-1,4-polyisoprene latex. Moreover, the spectra of NPs with and without latex coating exhibited an absorption peak at 573 cm^−1^, which corresponds to the metal-oxygen vibration, which is related to the vibration modes of intrinsic elongation of the M-O bond (Fe-O) at the tetrahedral site [15,69]. The interaction of biomolecules with oxide NPs may occur through -OH groups [70]. Since the MNPs and NRL are negatively charged, as revealed by the zeta-potential studies, electrostatic interaction should not be responsible for the adsorption of the NRL to the NPs’ surface [71]. Changes in the relative intensity of the peaks at 2960, 2920, and 2854 cm^−1^ and the displacement of the peak at 828 to 835 cm^−1^ related to the -CH_3_ stretching in the FTIR spectra of the particles containing the higher amount of NRL (NRL-400) compared to NRL reveals changes in the structure of the polymer due to adsorption onto the particle’s surface.

**Figure 5:**
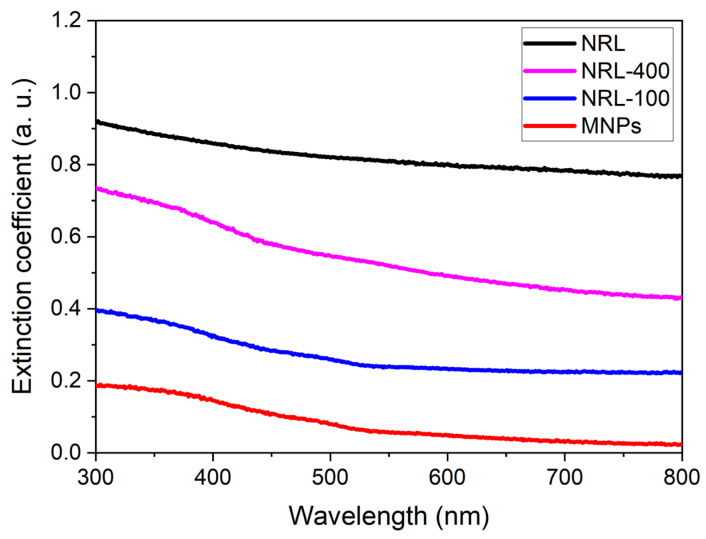
UV-Vi’s spectra of NRL, uncoated MNPs, and MNPs coated with different amounts of NRL.

UV-Vi’s absorbance spectra were acquired within the range of 300 to 800 nm and used to investigate the optical properties of NRL and the samples, as illustrated in Figure 5. The spectra obtained from the samples exhibited optical attenuation without pronounced absorption peaks related to the semi-metallic behavior of Fe_3_O_4_ NPs [72]. Extinction peaks can be observed at wavelengths around 390 nm, closely aligning with the characteristic wavelengths of IONPs [73,74,75]. The extinction values at 300 nm were 0.19, 0.40, and 0.73 (a. u.) for MNPs, NRL-100, and NRL-400, respectively. An increase in extinction was evident for the NRL-100 and NRL-400 samples compared to the uncoated sample. This increase may be associated with the amount of polymer used to coat the particles, which also influenced their sizes, as observed in the results obtained through DLS, DRX, and TEM [76].

**Figure 6:**
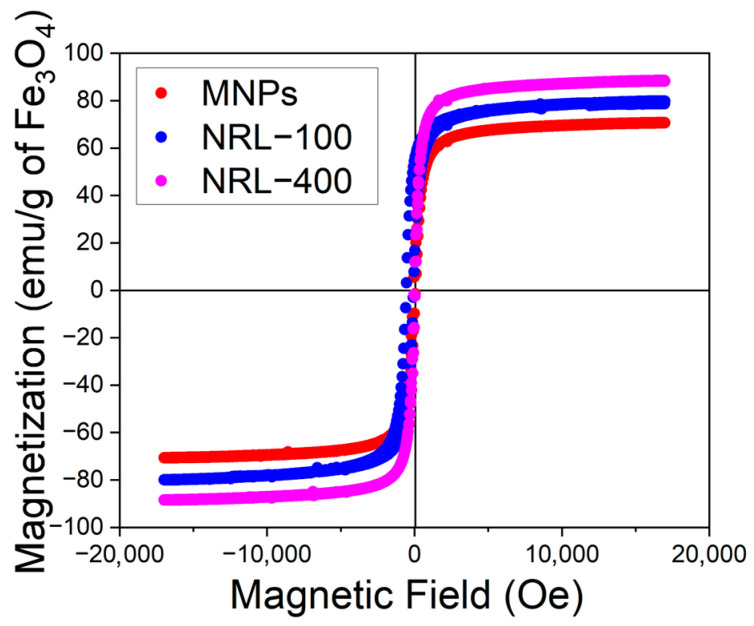
Magnetization curves after subtraction of the NRL mass from uncoated MNPs and MNPs coated with different amounts of NRL.

Figure 6 shows the room-temperature M-H curves of the samples under a maximum field amplitude of 2 T. NPs with diameters of 50 nm or less function as a single magnetic domain [25]. Iron oxides (maghemite and hematite) contain Fe^3+^ ions, and despite having magnetic properties (ferromagnetic and antiferromagnetic, respectively), after forming sufficiently small crystals, both become superparamagnetic [65].

The saturation magnetization (Ms) values obtained for the uncoated MNPs, NRL-100, and NRL-400, were 70, 62, and 59 emu/g (considering the total mass of uncoated MNPs and NRL-coated NPs), respectively. In addition, it showed low coercivity, with values of 36, 36.5, and 45 Oe, and residual magnetization of 4.22, 4.50, and 5 emu/g for the uncoated MNPs, NRL-100, and NRL-400, respectively. The reduction in Ms for NRL-100 and NRL-400 may be associated with the polymeric and magnetically inactive latex layer around the MNPs with an Ms close to zero [7,77,78]. Information obtained from the thermogravimetric analysis (TGA/DTGA) allows corrections (subtraction of the latex mass) in the magnetization curves of the uncoated MNPS, NRL-100, and NRL-400 [7,78]. See Figure S3 in the Supplementary Material for more information about TGA results. For uncoated MNPs, the curves exhibited a lower total mass loss, attributed to the evaporation of water or hydroxyl groups, both chemically and physically present on the surface of the NPs [79].

Results indicate that NRL-100 and NRL-400 undergo multistage degradation, featuring a primary peak and a smaller peak around it. According to Galiani [80], these peaks suggest the formation of thermally stable products, providing evidence of the multistage degradation in NRL [80]. In the temperature range of 200 to 270 °C, no loss of unsaturation related to rubber is observed. The first stage occurs in the range of 275 to 410 °C, where the curves show losses that are associated with the thermal degradation of isoprene, dipentene, and small amounts of p-mentene [81]. The second stage, which varies from 430 to 900 °C, is associated with the thermal decomposition of rubber carbon residues [80]. Other researchers have reported similar results [82,83]. The percentage of the mass loss for uncoated MNPS, NRL-100, and NRL-400 are around 2, 21, and 33%, respectively.

After subtracting the mass of the latex, it is observed that the Ms value increased. The corrected Ms values for uncoated MNPs, NRL-100 and NRL-400, were 70, 79, and 88 emu/g of Fe_3_O_4_, respectively. Figure S4 in the Supplementary Material shows the magnetization curves distinguishing emu/g (considering the total mass of the MNPs, which includes the MNPs core and the outer coating material (NRL)), emu/gFe_3_O_4_ (considering the mass corrected by thermogravimetric analysis, excluding the influence of the coating material (NRL), emphasizing only the core of the nanoparticles), and emu/g Fe (considering the mass corrected by elemental analysis (EDS), emphasizing only the iron present in the sample). This increase can be attributed to the influence of the binding polymer, which can decrease interparticle interactions, reducing the disorder of surface spins [84]. An increase in the magnetization of these MNPs is observed when reducing the mass of magnetically inactive polymers (NRL), suggesting that the presence of this material contributed to the formation of the particles [85]. Another factor that can affect magnetization is the size of NPs. According to the literature, the relationship between magnetization and particle size is attributed to a reduction in the effect of thermal fluctuations and magnetic disorder on the surface, resulting from a decrease in the surface-to-volume ratio [86,87,88]. In this specific case, NRL-400 exhibited a larger size than NRL-100 and MNPs, potentially leading to a higher Ms value.

### 3.2. Toxicity of the Nanoparticles to Cancer Cells

**Figure 7:**
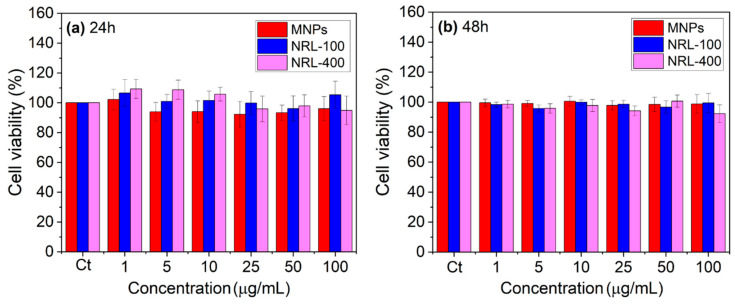
Cell viability of B16-F10 cells after incubation for (**a**) 24 h and (**b**) 48 h with varying concentrations of NPs.

The toxicity of NPs was assessed using a cell line of fusiform and epithelial cells isolated from the skin tissue of a mouse with melanoma (B16-F10). The results obtained after 24 and 48 h of treatment with the NPs are shown in Figure 7.

All concentrations of NPs exhibited viability above 80%, indicating non-cytotoxic under the tested conditions [89]. Similar results were obtained for the NRL-100 and NRL-400 samples. The physiological relevance of iron supports existing literature demonstrating the non-toxic nature of these NPs to the cells employed in this study [22,90]. In the case of latex-coated samples, their association with a colloidal system comprising proteins, lipids, and carbohydrates highlights their robust biocompatibility and bioactivity. This material, characterized by high angiogenic potential, offers flexibility, surface porosity, and good permeability, making it well-suited for several biomedical applications. Notably, it performs well in drug delivery systems, wound healing, and induction of tissue regeneration [91,92,93]. In addition, it was found that for certain concentrations, after 24 and 48 h of treatment with the NPs, cell viability values of over 100% were observed. This phenomenon is commonly associated with the process of hormesis, a biological response characterized by stimulating beneficial adaptive reactions at low doses of a substance or stressor, while high doses can be toxic or neutral [94,95]. The variability of the hormetic response depends on the concentration, the type of nanoparticle, and its surface properties. NRL-100 and NRL-400 NPs, particularly at lower concentrations, showed a significant increase in cell viability compared to uncoated MNPs. This effect is possibly due to the functional groups on the surface of the nanoparticles, which can amplify or mitigate the hormetic effect, resulting in different cell viability responses [96]. The low concentrations used were chosen based on studies already published to evaluate the initial performance of the nanoparticles [97,98,99]. Higher concentrations could be tested in the future, depending on the specific application requirements.

### 3.3. Theranostic Potential of NRL-Coated MNPs

**Figure 8:**
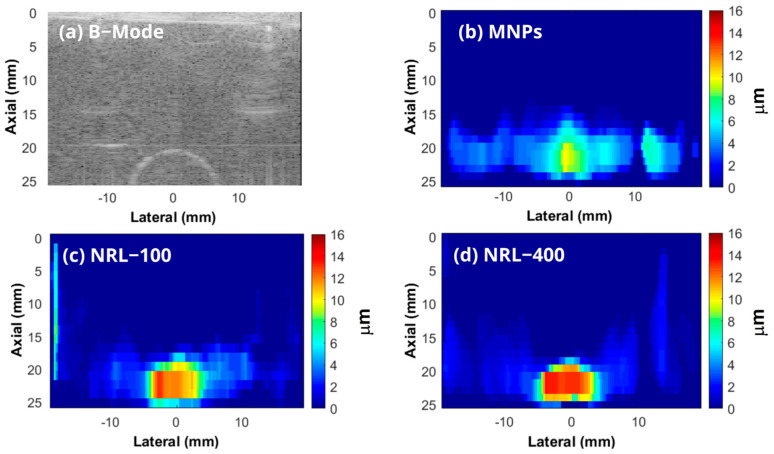
(**a**) B-Mode and MMUS image of the phantom containing (**b**) uncoated MNPs, (**c**) NRL-100, and (**d**) NRL-400.

Typical B-mode and MMUS images of the phantoms containing inclusions labeled with MNPs are shown in Figure 8. The regions in red show larger displacements indicating the locations of the NPs.

**Figure 9:**
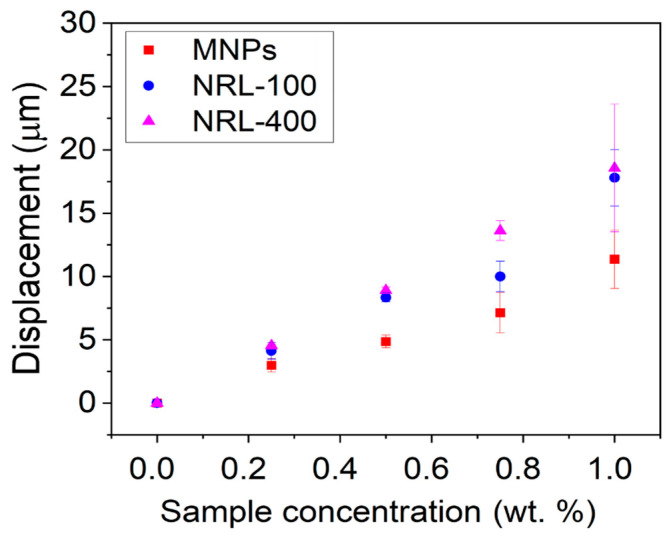
Average induced displacements within the inclusions for phantoms containing different concentrations of the produced NPs.

Figure 9 shows data collected from phantoms with varying weight concentrations of MNPs (1, 0.75, 0.50, and 0.25%) for uncoated MNPs, NRL-100, and NRL-400. The results show a direct correlation between the concentration of NPs and the displacements observed in the phantoms. Higher concentrations of NPs exhibited more significant displacements, and the amplitude of displacements demonstrated a linear increase in proportion to the nanoparticle concentration. However, even at low concentrations, the NPs showed a satisfactory magnetic response, resulting in expressive displacements, mainly for NRL-100 and NRL-400. For example, the average induced displacements within the inclusions for phantoms containing 0.50 wt.% of the uncoated MNPs, NRL-100, and NRL-400, were approximately 5 µm, 8 µm, and 9 µm, respectively, using a magnetic pulse width of 6 ms.

The magnetic force acting on the MNPs is linearly proportional to their magnetic susceptibility (χ), geometry (V), and structure [100]. Upon conducting a linear regression analysis on the results, coefficients of determination (R^2^): 0.95 for MNPs, 0.93 for NRL-100, and 0.99 for NRL-400 were obtained. These results suggested that a larger magnetic core size and higher susceptibility of MNPs led to a greater magnetic force generation, resulting in more pronounced displacements and increased contrast in the acquired images.

**Figure 10:**
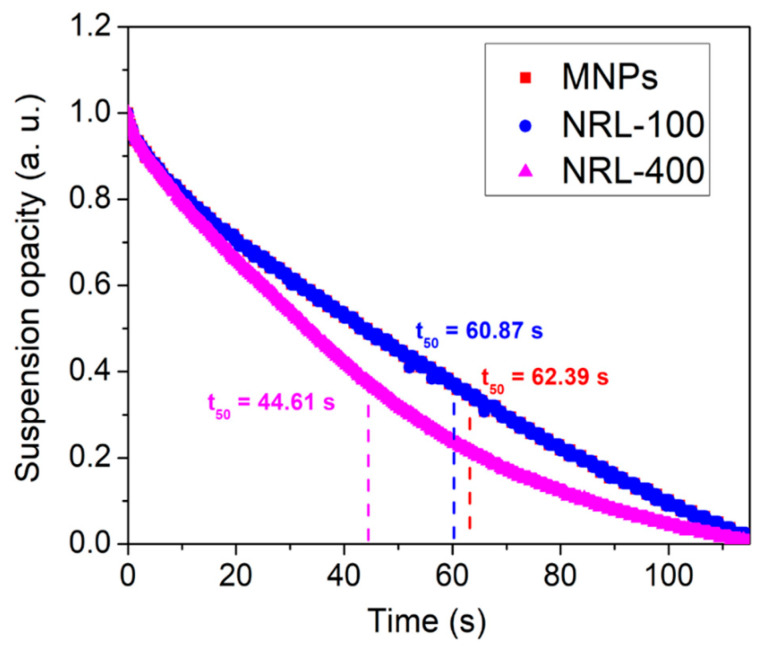
Magnetophoretic curves obtained for MNPs, NRL-100, and NRL-400.

Figure 10 provides relevant information on the behavior of MNPs (c = 0.50 wt.%) in a magnetic field gradient. The system includes an optical sensor that measures changes in the opacity of the sample over time during the process. The time required for the opacity of the sample to be reduced by 50%, known as t_50_, is a parameter widely used in the study of MNPs’ behavior.

The t_50_ results obtained through the magnetophoretic curves were 62.39, 60.87, and 44.61 s for the uncoated MNPs, NRL-100, and NRL-400, respectively. The attractive magnetic force increased with the size of NPs, resulting in elevated velocity of the latex-coated NPs towards the tube wall. These results are consistent with the findings of Olga Mykhaylyk et al. [101], who investigated particle acceleration, which depends on particle properties such as size and magnetization, concentration, field, and field gradient. Therefore, the results agree with those obtained through MMUS, indicating that NRL-100 and NRL-400 have superior magnetic properties compared to uncoated MNPs.

The results obtained through the magnetophoretic curves also correlate with the results obtained for zeta potential. NPs with higher colloidal stability (NRL-100 and NRL-400) tend to migrate faster in a magnetic field, resulting in a shorter magnetophoresis time. This correlation suggests that the coating that increases the zeta potential also influences the magnetic response of the particles, possibly modifying the interaction between the particles and the medium, allowing a more efficient magnetophoresis [102].

**Figure 11:**
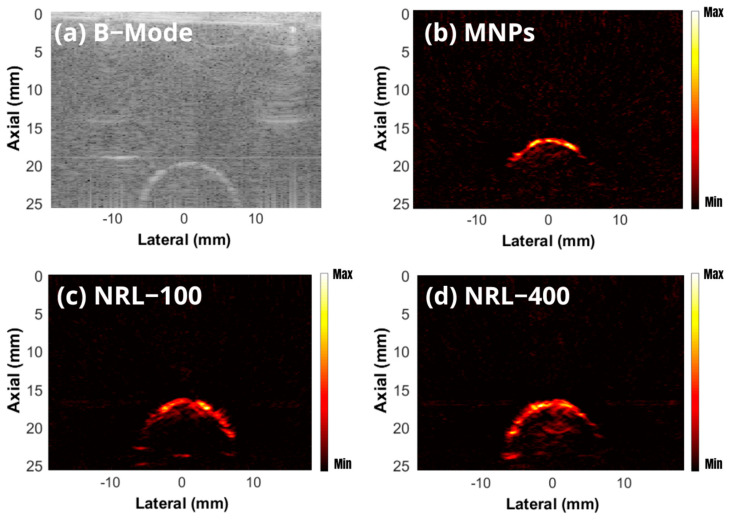
(**a**) Typical B-Mode image of a phantom. PA images at 750 nm of phantoms containing (**b**) uncoated MNPs, (**c**) NRL-100, and (**d**) NRL-400.

In assessing and optimizing PAI systems, it is common to use phantoms and samples of biological tissues such as chicken breast and pork tissue [103,104]. In this investigation, phantoms containing an inclusion labeled 0.50 wt.% of MNPs, NRL-100, and NRL-400, were used to evaluate their effectiveness as contrast agents for PAI. Figure 11a shows a typical B-mode image of a phantom, while Figure 11b–d shows PA images at 750 nm obtained from phantoms containing uncoated MNPs, (c) NRL-100, and (d) NRL-400, respectively.

It is evident that the quality of the PA images obtained from the phantom containing NRL-coated MNPS was improved compared to base MNPs, as illustrated in Figure 11b–d. The contrast values were 19.24 (0.91), 26.05 (1.23), and 28.81 (1.36) dB for phantom containing an inclusion of 0.50 wt.% of uncoated MNPs, NRL-100, and NRL-400, respectively. Coated NPs can increase the photoacoustic signal, leading to a significant improvement in image contrast. The SNR values were 24.72 (0.51), 31.44 (0.44), and 33.81 (0.46) dB for the phantoms containing an inclusion of 0.50 wt.% of uncoated MNPs, NRL-100, and NRL-400, respectively. In a noise-controlled scenario, such as that of the phantom, the contrast and SNR in PAI are directly related to the optical absorbance of the imaging target. Therefore, the increased contrast and SNR can be attributed to the higher extinction (as illustrated in Figure 5) of NRL-coated NPs in the near-infrared (NIR) region. This region of the electromagnetic spectrum presents low absorption and scattering properties of biological tissues, therefore enabling deeper light penetration and improved imaging depth, contributing to enhanced contrast in PAI [105].

**Figure 12:**
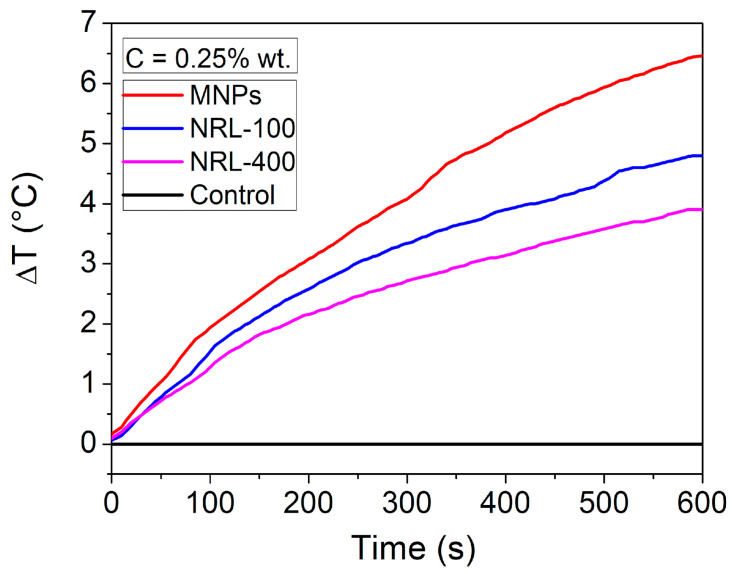
Temperature variation as a function of time for the samples produced.

The potential of MNPs in MH was evaluated through calorimetric measurements. Samples with varying concentrations (0.25, 0.50, 0.75, 1, 1.25, and 1.50 wt.%) along with a water control were subjected to an external magnetic field with a frequency of 137 kHz and an amplitude of 100 Oe. As an illustrative example, Figure 12 shows the temperature variation as a function of time for all samples of 0.25 wt.%.

**Figure 13:**
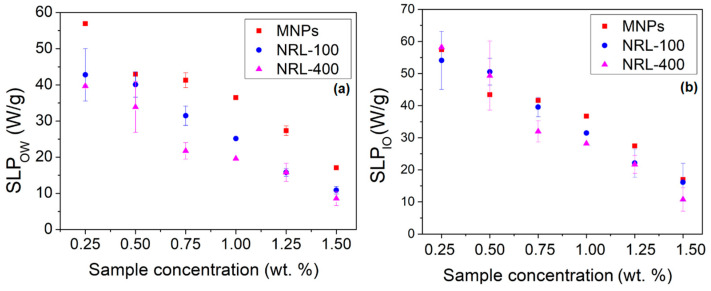
(**a**) The SLP_OW_ values of the samples at different concentrations by wt.% of NPs considering the overall weight of the coated MNPs, which includes both the MNPs core and the outer coating material, and (**b**) the SLP_IO_ values measured as a function of TGA-corrected weight concentrations for the produced nanoparticles, focusing exclusively on the nanoparticle core.

SLP was determined using Equation (3), and the temperature–time results were fitted to the Box–Lucas equation [59]. Figure 13a illustrates the SLP values considering the overall weight of the nanoparticles (SLP_OW_), which includes both the core and the outer coating material, plotted against the weight concentrations of MNPs. The findings depicted in Figure 13a demonstrate a reduction in SLP_OW_ with escalating sample concentrations. A linear adjustment was carried out for the results, resulting in the following coefficients of determination (R^2^): 0.94 for MNPs, 0.98 for NRL-100, and 0.98 for NRL-400. This phenomenon can be ascribed to the heightened particle agglomeration, potentially impeding relaxation mechanisms [106]. When the sample concentrations decrease, the NPs become more diluted, which results in less aggregation of MNPs and an increase in the average distance between them. The dilution decreased the dipolar interaction between NPs, which allowed them to regain their ability to heat the surrounding medium [107]. According to the results, well-dispersed NPs are expected to have higher SLP_OW_ values and higher heat transfer coefficients, indicating better heat generation performance. However, it is worth mentioning that the Neel and Brownian mechanisms can significantly generate thermal energy from magnetic energy [108].

NRL-100 and NRL-400 samples exhibited lower SLP_OW_ values compared to the uncoated MNPs. These NPs are incorporated and physically confined within the latex matrix; a small interparticle separation is observed, approximately equal to the diameter of a single particle. This condition hinders the thermal activation of the system in a low AC field, where the NPs need to overcome the dipole field produced by discrete NPs. Other authors have reported similar results [109]. Consequently, the NRL-100 and NRL-400 NPs showed reduced performance in the production of hyperthermia when compared to uncoated MNPs, as indicated by the results obtained in the present study. According to Usov et al., there are two distinct modes of heating, called viscous and magnetic modes, in which Brownian and Néel relaxation mechanisms are predominant, respectively [110]. Usov and his team quantified the modes in terms of field strengths and concluded that viscous forces predominate only at low field strengths (100 Oe). In general, all investigations carried out demonstrated that the Brownian relaxation mechanism is influenced by both the viscosity of the medium and the field strength, which leads to significant impacts on the global SLP values and may result in a scenario in which only Néel relaxation contributes to the heat dissipation mechanism [110,111]. Consequently, the NRL-100 and NRL-400 samples showed reduced performance in producing hyperthermia when compared to uncoated MNPs, as indicated by the results obtained.

On the other hand, the mass corrected via thermogravimetric analysis excludes the influence of the coating material, giving exclusive emphasis to the iron oxide nanoparticle core. This comparison can determine the real impact of the coating on the heat generation capacity of the NPs. Using Equation (3) and considering only the iron mass of the produced NPs (MNPs, NRL-100, and NRL-400), duly corrected according to the TGA, the result for the SLP_IO_ is obtained, as shown in Figure 13b.

By considering the total mass of the coated NPs, it is possible to obtain an assessment of the SLP of the complete system, including all contributions from the materials involved. A linear adjustment was carried out, resulting in the following coefficients of determination (R^2^): 0.96 for MNPs, 0.99 for NRL-100, and 0.99 for NRL-400. On the other hand, by comparing the SLP_OW_ variation considering the total mass of the NPs (Figure 13a) and that corrected by TGA (Figure 13b), it can be confirmed that coating in a viscous matrix hinders the thermal activation of the NPs in a low alternating current field, consequently reducing the performance of these NPs for magnetic hyperthermia applications.

## 4. Conclusions

In conclusion, this study focused on the synthesis, characterization, and diverse applications of MNPs coated with varying amounts of NRL derived from Hevea brasiliensis. Characterization techniques, including XRD and TEM, affirmed the small sizes of the nanoparticles. Magnetization curves showed that the NRL-coated MNPs exhibited a higher magnetization than their uncoated counterparts. Additionally, zeta potential measurements indicated enhanced colloidal stability for NRL-coated MNPs. The biological evaluation demonstrated low toxicity to cancer cells, reinforcing the potential of these MNPs for biomedical applications. The study explored their performance in diverse techniques, including MMUS, PAI, and MH. In MMUS, NRL-coated samples (NRL-100 and NRL-400) displayed more significant displacements and superior image contrasts than uncoated MNPs. PAI results revealed improved signal-SNR and contrast for NRL-coated samples, highlighting their potential as contrast agents. Calorimetric measurements for MH showcased the heat-generating capacity of NRL-coated MNPs. The findings collectively suggest that MNPs coated with NRL, especially NRL-100 and NRL-400, exhibit favorable physicochemical properties, making them promising candidates for various biomedical applications. Although the NRL-coated MNPs showed a lower SLP, their performance in MMUS, PAI, and MH still demonstrates their versatility and potential for future diagnostic and therapeutic applications.

## Data Availability

The original contributions presented in the study are included in the article/Supplementary Materials, further inquiries can be directed to the corresponding author.

## Supplementary Materials

The following supporting information can be downloaded at: https://www.mdpi.com/article/10.3390/pharmaceutics16111474/s1, Figure S1: Hydrodynamic diameter of the NRL, MNPs, NRL-100 and NRL-400; Figure S2: SEM image and table containing the elemental results obtained by EDS; Figure S3: Thermogravimetric analysis curves (TGA) and its derivatives (DTGA) for samples (a) MNPs, (b) NRL-100 and (c) NRL-400; Figure S4: Magnetization curves for the samples produced distinguishing (a) emu/g considering the total mass of MNPs, which includes the MNPs core and the external coating material (NRL); (b) emu/g Fe_3_O_4_ considering the mass corrected by thermogravimetric analysis, excluding the influence of the coating material (NRL), emphasizing only the nanoparticle core; and emu/g Fe considering the mass corrected by elemental analysis (EDS), emphasizing only the iron present in the sample.
