# Supplementary material for: Improving the Theranostic Potential of Magnetic Nanoparticles by Coating with Natural Rubber Latex for Ultrasound, Photoacoustic Imaging, and Magnetic Hyperthermia: An In Vitro Study
Source: Pharmaceutics. 2024 Nov 19;16(11):1474. doi: 10.3390/pharmaceutics16111474 (PMC11597301; doi:10.3390/pharmaceutics16111474)
Supplement: Supplementary file 1 [file pharmaceutics-16-01474-s001.zip › pharmaceutics-3209371-supplementary.pdf]

Improving the Theranostic Potential of Magnetic Nanoparticles by Coating with Natural Rubber Latex for Ultrasound, Photoacoustic Imaging, and Magnetic Hyperthermia: An In Vitro Study

Thiago T. Vicente [1], Saeideh Arsalani [1,2], Mateus S. Quiel [1], Guilherme S. P. Fernandes [1], Keteryne R. da Silva [3], Sandra Y. Fukada [3], Alexandre J. Gualdi [4], Éder J. Guidelli [1], Oswaldo Baffa [1], Antônio A. O. Carneiro [1], Ana Paula Ramos [5] and Theo Z. Pavan [1,*]

1 Department of Physics, FFCLRP, University of São Paulo, Av. Bandeirantes 3900, Ribeirão Preto 14040-901, São Paulo, Brazil; thiagotiburcio37@usp.br (T.T.V.); saeideh.arsalani@utsouthwestern.edu (S.A.); setubalmateus@usp.br (M.S.Q.); guilherme.santos.fernandes@usp.br (G.S.P.F.); guidelli@usp.br (É.J.G.); baffa@usp.br (O.B.); adilton@usp.br (A.A.O.C.)
2 UT Southwestern Medical Center, Biomedical Engineering Department, Dallas, TA 75235-7323, USA
3 Department of BioMolecular Sciences, FCFRP, University of São Paulo, Av. Professor Doutor Zeferino Vaz, sn, Ribeirão Preto 14040-901, São Paulo, Brazil; keteryne@alumni.usp.br (K.R.d.S.); sfukada@usp.br (S.Y.F.)
4 Department of Physics, Federal University of São Carlos, Rod. Washington Luiz, km 235, São Carlos 13565-905, São Paulo, Brazil; gualdi@df.ufscar.br
5 Department of Chemistry, FFCLRP, University of São Paulo, Av. Bandeirantes 3900, Ribeirão Preto 14040-901, São Paulo, Brazil; anapr@ffclrp.usp.br
* Correspondence: theozp@usp.br; Tel.: +55-16-33153780

The hydrodynamic diameter of MNPs was determined using DLS. Measurements were conducted on a colloidal suspension of pure NRL and on Fe_3O_4 nanoparticles, both with and without NRL coating, after washing by magnetic separation with deionized water. The results obtained after washing can be seen in Figure S1.

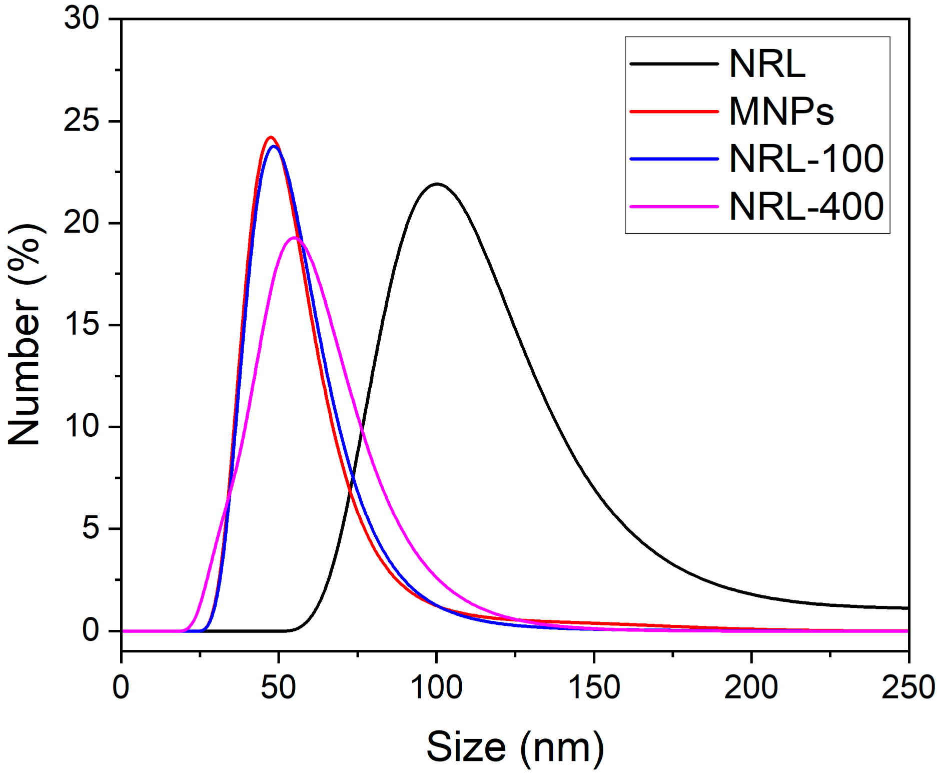

Figure S1 - Hydrodynamic diameter of the NRL, MNPs, NRL-100 and NRL-400.

The ZEISS-EVO 50 scanning electron microscope (SEM) can be used to analyze the micro and nanostructural characteristics of solid samples. By using a high-energy electron beam (primary beam), the equipment makes it possible to obtain images with a three-dimensional appearance and high surface resolution. In addition to the image formation process, the formation of X-rays emitted by the sample and their detection enables elemental microanalysis (EDS) to be carried out. Figure S2 shows an SEM image of the MNPs, accompanied by a table with the values obtained for the composition of the material produced.

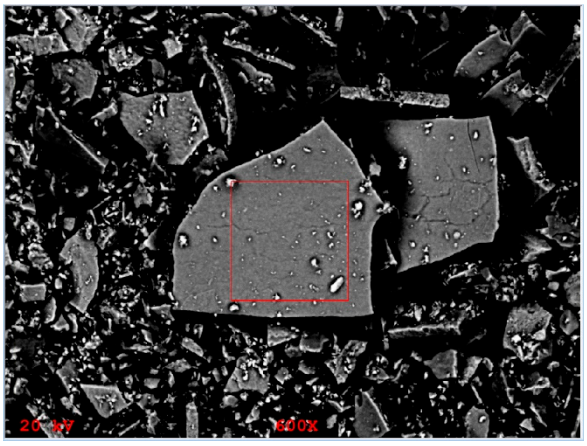

Elt.	Line	Intensity (c/s)	Atomic %	Conc	Units	
O	Ka	1,601.67	51.838	23.568	wt.%	
Fe	Ka	5,844.49	48.162	76.432	wt.%	
			100.000	100.000	wt.%	Total

kV	20.0
Takeoff Angle	35.0°
Elapsed Livetime	100.0

Figure S2 - SEM image and table containing the elemental results obtained by EDS.

To evaluate the stability and physical-chemical properties of the compound, thermogravimetric analysis (TGA) and its derivative (DTGA), percentage of mass loss as a function of temperature, were carried out. For this, powdered samples were used and heated up to 900 °C with a heating rate of 10 °C/min in an oxidizing atmosphere. Figure S4 shows the TGA/DTGA curves related to total mass loss for samples (a) MNPs, (b) NRL-100 and (c) NRL-400.

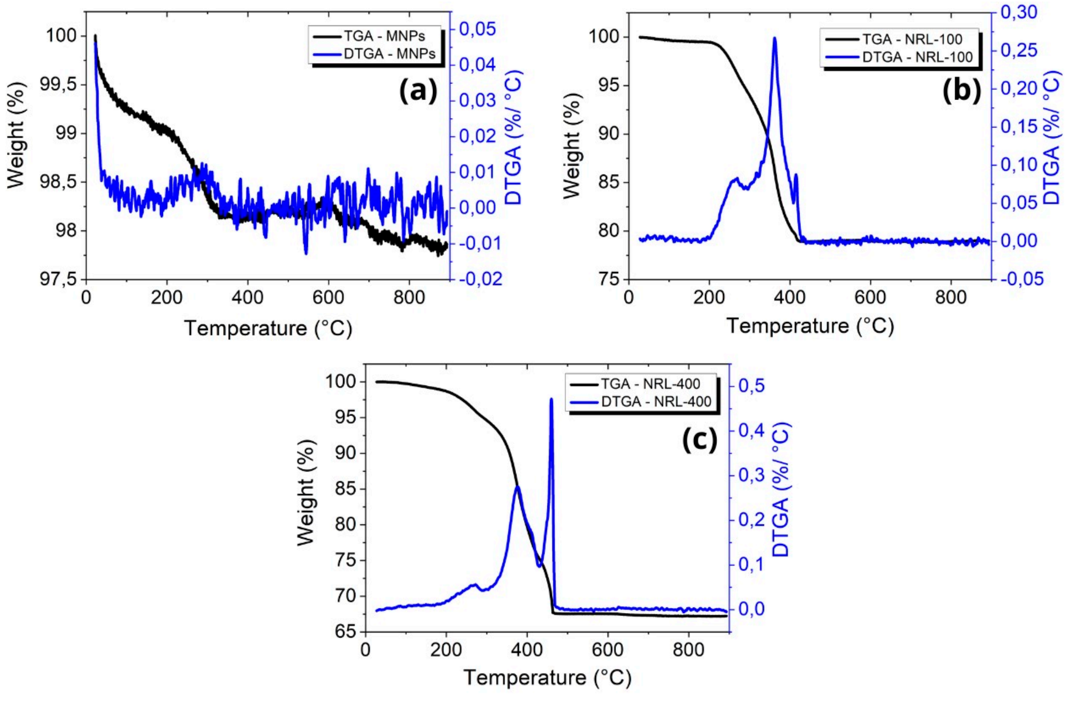

Figure S3 - Thermogravimetric analysis curves (TGA) and its derivatives (DTGA) for samples (a) MNPs, (b) NRL-100 and (c) NRL-400.

Figure S4 shows the magnetization curves (a) considering the total mass of MNPs, which includes both the MNP core and the external coating material, (b) the mass corrected by thermogravimetric analysis excluding the influence of the coating material, emphasizing only the nanoparticle core, and (c) the mass corrected by elemental analysis (EDS), emphasizing only the iron present in the sample.

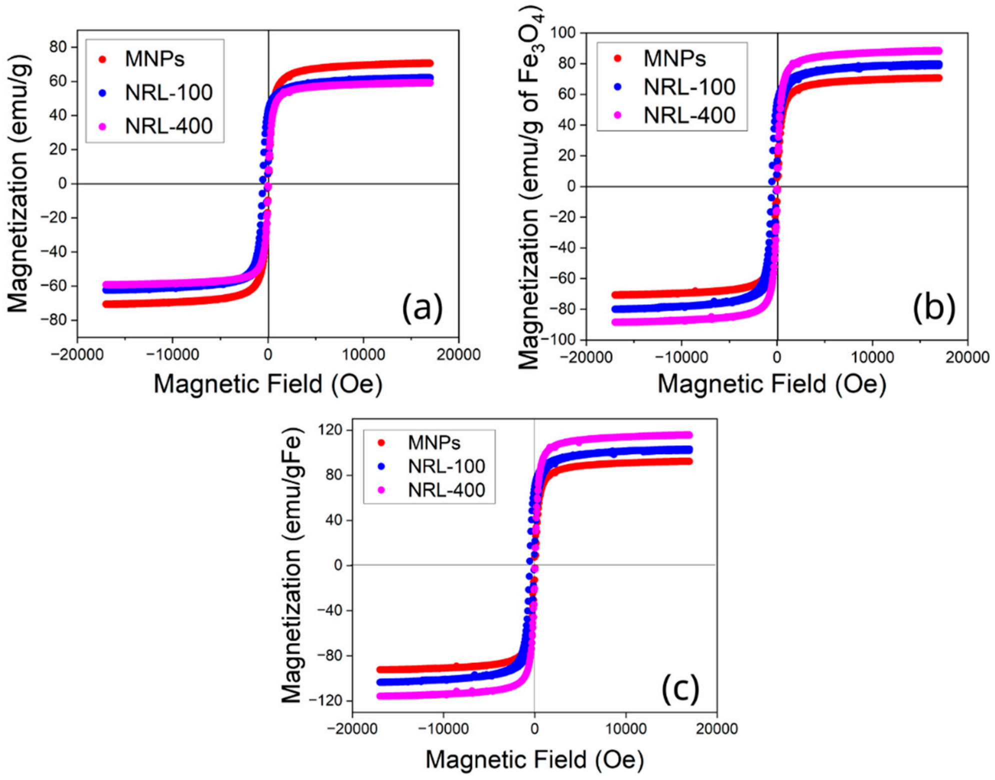

Figure S4 – Magnetization curves for the samples produced distinguishing (a) emu/g considering the total mass of MNPs, which includes the MNPs core and the external coating material (NRL)); (b) emu/gFe_3O_4 considering the mass corrected by thermogravimetric analysis, excluding the influence of the coating material (NRL), emphasizing only the nanoparticle core); and emu/g Fe (considering the mass corrected by elemental analysis (EDS), emphasizing only the iron present in the sample.
